# Admission and Readmission/Death Patterns in Hospitalized and Non-hospitalized First-Ever-in-a-Lifetime Stroke Patients During the First Year: A Population-Based Incidence Study

**DOI:** 10.3389/fneur.2021.685821

**Published:** 2021-09-08

**Authors:** Pedro Abreu, Rui Magalhães, Diana Baptista, Elsa Azevedo, Manuel Correia

**Affiliations:** ^1^Department of Neurology, Centro Hospitalar Universitário de São João, Porto, Portugal; ^2^Department of Clinical Neurosciences and Mental Health, Faculdade de Medicina, Universidade do Porto, Porto, Portugal; ^3^Instituto de Ciências Biomédicas Abel Salazar, Universidade do Porto, Porto, Portugal; ^4^Department of Neurology, Hospital Santo António – Centro Hospitalar Universitário do Porto, Porto, Portugal

**Keywords:** stroke, epidemiology, community-based study, patient admission, stroke readmissions, mortality, outcome

## Abstract

**Background:** Hospitalization and readmission rates after a first-ever-in-a-lifetime stroke (FELS) are considered measures of quality of care and, importantly, may give valuable information to better allocate health-related resources. We aimed to investigate the hospitalization pattern and the unplanned readmissions or death of hospitalized (HospS) and non-hospitalized stroke (NHospS) patients 1 year after a FELS, based on a community register.

**Methods:** Data about hospitalization and unplanned readmissions and case fatality 1 year after a FELS were retrieved from the population-based register undertaken in Northern Portugal (ACIN2), comprising all FELS in 2009–2011. We used the Kaplan–Meier method to estimate 1-year readmission/death-free survival and Cox proportional hazard models to identify independent factors for readmission/death.

**Results:** Of the 720 FELS, 35.7% were not hospitalized. Unplanned readmission/death within 1 year occurred in 33.0 and 24.9% of HospS and NHospS patients, respectively. The leading causes of readmission were infections, recurrent stroke, and cardiovascular events. Stroke-related readmissions were observed in more than half of the patients in both groups. Male sex, age, pre- and post-stroke functional status, and diabetes were independent factors of readmission/death within 1 year.

**Conclusion:** About one-third of stroke patients were not hospitalized, and the readmission/death rate was higher in HospS patients. Still, that readmission/death rate difference was likely due to other factors than hospitalization itself. Our research provides novel information that may help implement targeted health-related policies to reduce the burden of stroke and its complications.

## Introduction

Nowadays, there is a general consensus that most stroke patients should be hospitalized to have access to specialized, high-quality, evidence-based stroke interventions, thus increasing their chances of survival, improving their functional outcome, and preventing stroke recurrence (1–4). Despite this evidence, 12–20% of stroke patients are not hospitalized (4).

Readmissions are currently a measure of the hospital's performance and quality of care (5), despite some well-characterized limitations (6). Readmissions after stroke are common, particularly in the first 30 days, ranging from 6 to 21% (7).

The literature has vast information on readmission and mortality after an initial stroke hospitalization. Nonetheless, except for some particular types of stroke, such as transient ischemic attack and minor ischemic stroke (IS), few studies use population-based data to study how non-hospitalized stroke (NHospS) patients compare to hospitalized stroke (HospS) patients in terms of readmission after stroke (4, 8, 9). Studying such differences may uncover important data that may help implement novel health policies regarding the allocation of stroke-related resources and public research funding.

We aimed to investigate the hospitalization pattern and its relevance, the overall 1-year readmission/death rate, and the incidence, causes, and risk factors of unplanned readmission or death of HospS and NHospS patients, based on a Portuguese community register of first-ever-in-a-lifetime stroke (FELS) patients presenting to the emergency department (ED).

## Methods

Data were obtained from the second population-based register undertaken in Northern Portugal (ACIN2), comprising all FELS recorded between October 2009 and September 2011 in the population registered in the Western Porto Health Centers Group (about 190,000 persons) and two health centers in rural regions in Northern Portugal (Mirandela and Vila Pouca de Aguiar, involving about 46,000 persons) (10). We used the unique National Health Service (NHS) number to identify the study population. We only analyzed data of patients from Porto, and the Ethics Committee of the Centro Hospitalar Universitário de São João and the Centro Hospitalar Universitário do Porto approved this study.

Multiple sources of information were used to identify all patients with a FELS. Hot-pursuit and cold-pursuit ascertainment involving community-based and hospital-based information sources were considered (10). Hot pursuit encompassed a daily review of emergency admissions and referrals to the project outpatient clinic at Hospital Santo António—Centro Hospitalar Universitário do Porto. Cold pursuit was used to check for completeness of hot-pursuit identification (11). Patients were examined as soon as possible after symptom onset at the ED during their hospital stay or at the project outpatient clinic within 1 month and were then followed up until 3 months after stroke. More detailed information is described elsewhere (10). Information about readmissions after the 3-month follow-up period was collected retrospectively. For readmission and death identification, a record-linkage methodology was used, and all clinical information was retrieved from clinical records.

The World Health Organization “stroke” definition and Sudlow's and Warlow's stroke pathological types—IS, intracerebral hemorrhage (ICH), and subarachnoid hemorrhage (SAH)—were considered for the corresponding concepts (12, 13). All patients performed a brain computerized tomography and/or magnetic resonance imaging to confirm and define pathological stroke types. TOAST criteria and Bamford Oxfordshire classification were used to define IS etiology and clinical syndromes (14, 15). Stroke severity at the first medical evaluation was characterized as mild, moderate, or severe based on the National Institutes of Health Stroke Scale (NIHSS) (12) (NIHSS ≤ 7, 8–16, or ≥17, respectively), except for SAH. Whenever NIHSS was unavailable, the score was estimated retrospectively from the patients' clinical records, if valid for that purpose (16).

Regarding the patients' care, the following factors were registered: time from symptom onset to ED, inpatient admission, and length of stay. Stroke patients were identified as “hospitalized” when they were hospitalized due to a FELS or when a FELS occurred while already in the hospital. Stroke patients were identified as “non-hospitalized” when they were not hospitalized after a FELS, including patients that were managed at the NHS ED only, primary care outpatient clinics, private outpatient clinics, or EDs.

Pre- and post-stroke (at hospital admission) functional status was assessed with the modified Rankin Scale (mRS) (17). Pre-stroke disability was defined as having an mRS score >1.

The following criteria were considered as pre-stroke risk factors: (a) previous diagnosis/treatment of hypertension; (b) previous diagnosis/treatment of diabetes mellitus or fasting glycemia >126 mg/dl, post-prandial glycemia ≥200 mg/dl, and/or ≥200 mg/dl in the 2-h glucose tolerance test; (c) atrial fibrillation in electrocardiogram or documented in the patient's records; (d) previous diagnosis/treatment of hypercholesterolemia; (e) history of myocardial infarction; and (f) current smoking habits (in the preceding 12 months) (10). Other pre-stroke comorbidities such as congestive heart failure, dementia, HIV infection, and malignant neoplasm were included after reviewing patients' medical records using the International Classification of Diseases 9^th^ Revision (*ICD-9*) diagnosis code.

Planned readmissions were defined as readmissions to perform a scheduled procedure (e.g., cranioplasty) and planned hospitalizations (e.g., rehabilitation) (18). Unplanned readmissions were defined as >24-h hospitalizations due to unexpected causes and emergency episodes leading to death that did not fulfill any planned readmission criterion (18). Unplanned readmissions after a planned admission were also acknowledged. Only unplanned readmissions within 1 year of the index hospitalization or index ED episode were considered. Patients who died during their evaluation in the ED or index hospitalization were excluded since they could not be readmitted.

Two neurology investigators reviewed the patients' medical records using the *ICD-9* diagnosis code to obtain and validate the unplanned readmission causes. The main diagnosis-related unplanned readmission group code was identified for statistical data and subgroup analyses. Stroke-related readmissions were defined as recurrent vascular events and complications that warranted readmission, including stroke, pneumonia, urinary tract infection, peripheral and coronary artery disease, hip fracture, and pulmonary embolism (19, 20). A composite outcome event of unplanned first-ever readmission or death without readmission within 1 year after stroke was considered to capture all negative health outcomes (5).

### Statistics

Sociodemographic characteristics were summarized using descriptive statistics. The baseline and clinical characteristics of HospS and NHospS were compared using the chi-square test or the Fisher exact test for categorical variables and the *t*-test or the Mann–Whitney *U*-test for continuous variables. The normality of distributions was assessed using the Shapiro Wilk test. The overall cumulative readmission/death-free survival over 12 months was estimated using the Kaplan–Meyer method. Independent factors for readmission were evaluated using Cox proportional hazard models. A two-sided *p*-value of < 0.05 was considered statistically significant in all analyses.

Data analysis was performed using SPSS Statistics v24.

## Results

### Study Cohort

**Figure 1:**
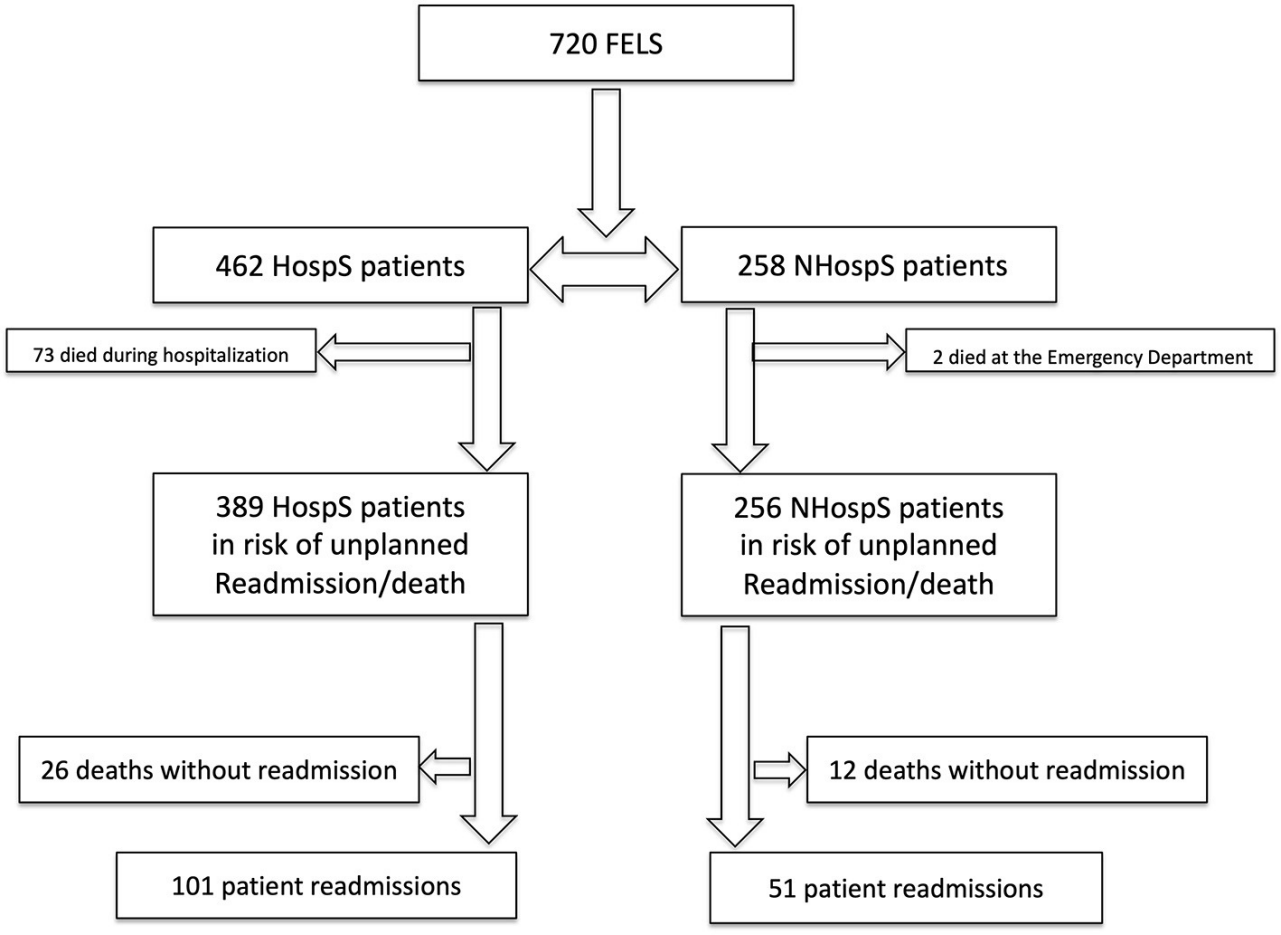
Study design for cohort follow-up. FELS, first-ever-in-a-lifetime stroke; HospS, hospitalized stroke patients; NHospS, non-hospitalized stroke patients.

Of an initial cohort of 720 FELS patients in the ACIN2 database, 35.7% were not hospitalized and were managed in the community. Reasons for non-hospitalization are depicted in Table 1. Since two patients died in the ED and 73 patients died during their index hospitalization, 645 FELS patients were at risk of an unplanned readmission/death (Figure 1). Patients had a mean age of 71 years, and 359 (55.7%) were women. All patients performed brain imaging at acute phase.

**Table 1:** Reasons for non-hospitalization of stroke patients (*n* = 258).

	***n***	**%**
Dead while in ED	2	0.8
Patient hospitalization refusal/ED abandonment	7	2.7
Community managed/did not attend to ED	10	3.9
Patient already institutionalized in care facilities	7	2.7
Medical/family decision	11	4.3
Time to ED medical assistance >48 h^*^	35	13.6
NIHSS <5	168	65.1
NIHSS 0–2	119	46.1
NIHSS 3–4	49	19.0
NIHSS 5–9	10	3.9
Other diagnosis^‡^	8	3.1

*ED, emergency department; NIHSS, National Institutes of Health Stroke Scale.*

* *Includes 26 with an NIHSS <5.*

† *Includes 59 other diagnoses later confirmed as stroke.*

‡ *Later confirmed as stroke*.

Comparing the baseline characteristics of HospS and NHospS patients at risk of readmission/death (Table 2), HospS patients were younger (70.2 vs. 72.7, *p* = 0.033); had more cases of cardiac disease (39.3 vs. 30.1%, *p* = 0.016), particularly atrial fibrillation (23.9 vs. 12.5%, *p* < 0.001); and had more hemorrhagic strokes (*p* < 0.001). The HospS patients group also had more total anterior circulation infarcts and fewer lacunar infarcts (*p* < 0.001), more large-artery atherosclerosis and cardioembolic strokes, and fewer small vessels or undetermined/not investigated strokes (*p* < 0.001).

**Table 2:** Baseline characteristics of first-ever-in-a-lifetime stroke patients in risk of readmission.

			**Hospitalized**				
	**Overall**		**Yes**		**No**		
	**(** ***n*** **=** **645)**		**(** ***n*** **=** **389)**		**(** ***n*** **=** **256)**		
	***n***	**%**	***n***	**%**	***n***	**%**	***p*-Value**
Women	359	55.7	208	53.5	151	59.0	0.168
Mean age (SD), years	71.2	(14.7)	70.2	(15.1)	72.7	(13.9)	**0.033**
<45	37	5.7	29	7.5	8	3.1	0.080
45–64	163	25.3	100	25.7	63	24.6	
65–84	326	50.5	195	50.1	131	51.2	
≥85	119	18.4	65	16.7	54	21.1	
Pre-existing comorbidities							
Hypertension	495	76.7	297	76.3	198	77.3	0.770
Diabetes mellitus	167	25.9	102	26.2	65	25.4	0.814
Cardiac disease^*^	230	35.7	153	39.3	77	30.1	**0.016**
Atrial fibrillation	125	19.4	93	23.9	32	12.5	** <0.001**
Myocardial infarction	59	9.1	39	10.0	20	7.8	0.340
Congestive heart failure	123	19.1	74	19.0	49	19.1	0.970
Hypercholesterolemia	309	47.9	182	46.8	127	49.6	0.483
Smoking	229	35.5	144	37.0	85	33.2	0.322
Dementia	79	12.2	45	11.6	34	13.3	0.516
Neoplasm	89	13.8	60	15.4	29	11.3	0.140
HIV	8	1.2	6	1.5	2	0.8	0.143
Pre-stroke mRS≥2	201	31.2	130	33.4	71	27.7	0.127
Stroke pathological type							** <0.001**
Ischemic stroke	563	81.5	317	81.5	246	96.1	
Intracerebral hemorrhage	65	10.1	58	14.9	7	2.7	
Subarachnoid hemorrhage	17	2.6	14	3.6	3	1.2	
Ischemic stroke subtype							** <0.001**
Total anterior circulation infarct	87	15.5	77	24.3	10	4.1	
Partial anterior circulation infarct	171	30.4	104	32.8	67	27.2	
Lacunar infarct	166	29.5	62	19.6	104	42.3	
Posterior circulation infarct	139	24.7	74	23.3	65	26.4	
Ischemic stroke etiology							** <0.001**
Large-artery atherosclerosis	70	12.4	49	15.5	21	8.5	
Cardioembolism	135	24.0	103	32.5	32	13.0	
Small-artery occlusion	130	23.1	51	16.1	79	32.1	
Other determined/more than one cause	23	4.1	14	4.4	9	3.7	
Undetermined/not investigated	205	31.8	100	25.7	106	41.4	
Median NIHSS (IQR)^†^	4	(1-8)	5	(2-13)	2	(1-4)	** <0.001**
Post-stroke mRS≥2 (at hospital admission)	513	79.5	339	87.1	174	68.0	** <0.001**
Medical attention							** <0.001**
<3 h	257	39.8	198	50.9	59	23.0	
3–24 h	243	37.3	142	36.5	101	39.5	
>24 h	145	22.5	49	12.6	96	37.5	
Previous admissions	59	9.1	46	11.8	13	5.1	**0.012**

*SD, standard deviation; mRS, modified Rankin Scale; IQR, interquartile range.*

* *Includes atrial fibrillation, myocardial infarction, arrhythmia, and congestive heart failure.*

† *Excluding subarachnoid hemorrhages.*

*Bold entries indicate statistical significance (p < 0.05)*.

The NIHSS median score was higher in HospS patients (5 vs. 2, *p* < 0.001), as was post-stroke disability (mRS ≥ 2, 87.1 vs. 68.0%, *p* < 0.001). Hospitalized stroke patients also had a higher likelihood of having had a previous admission (11.8% vs. 5.1, *p* = 0.012).

Overall, 500 (77.1%) patients sought medical assistance within 24 h of stroke symptoms onset. Of those seeking medical attention in this time interval, HospS patients were more likely to arrive in the first 3 h after stroke (*p* < 0.001). We found no differences between HospS and NHospS patients regarding other baseline characteristics. The median hospital length of stay was 9 days (interquartile range: 4–19).

### Overall Unplanned Readmission and Mortality Without Readmission

Overall, 127 HospS patients and 63 NHospS patients were readmitted/deceased within 1 year: 101 (79.5%) unplanned readmissions and 26 (20.5%) deaths without readmission in the HospS patient's cohort, and 51 (80.9%) unplanned readmissions and 12 (19.0%) deaths without readmission in the NHospS patient's cohort (Figure 1).

### Readmission or Death Rates

**Figure 2:**
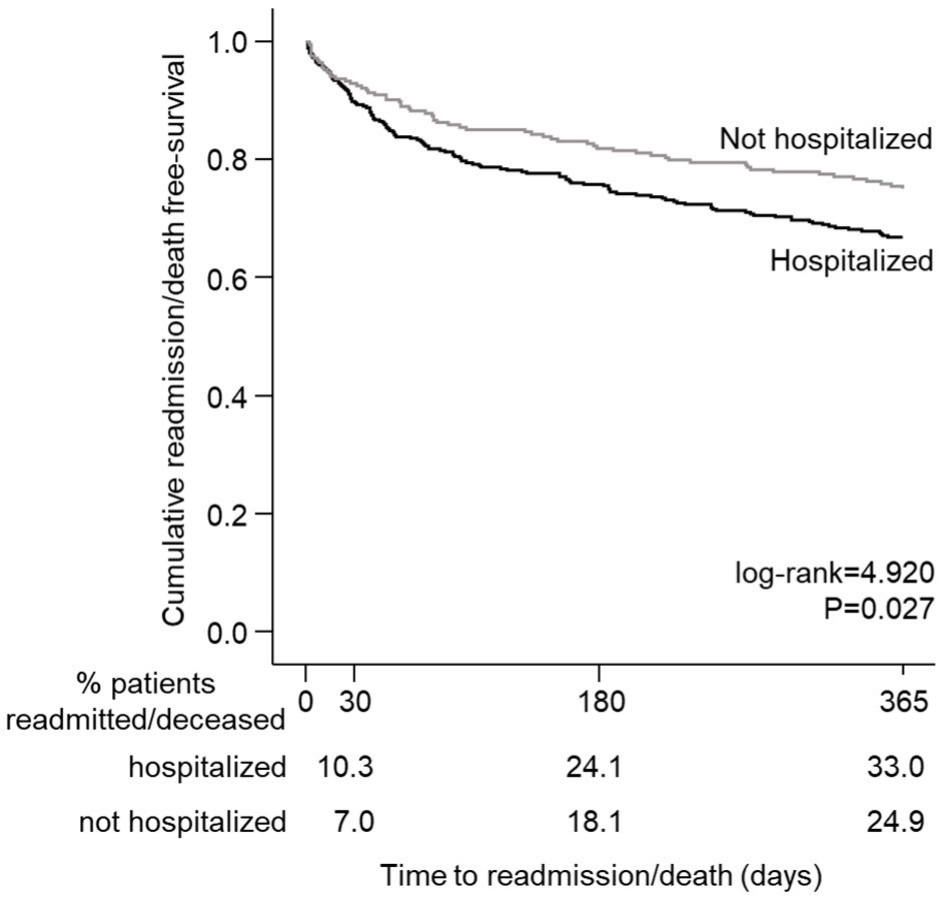
Kaplan–Meier survival curve showing a stroke patient's probability of remaining free of readmission/death after discharge.

Figure 2 shows the cumulative readmission/death-free survival. Hospitalized stroke patients were more likely to be readmitted or deceased within 1 year than NHospS patients (p = 0.028). The all-cause readmission/death rate at 30, 180, and 1 year in the HospS patients was 10.3, 24.1, and 33.0%, respectively, and in the NHospS patients, it was 7.0, 18.1, and 24.9%, respectively.

### Readmission Causes and Characterization

Table 3 summarizes patient characteristics and the reasons for hospital readmissions within the first year. Although not significant, the readmission rate was higher in HospS than in NHospS (26.0 vs. 19.9%, p = 0.077). The median time for readmission was 64 days (interquartile range: 22–184 days) in HospS patients and 78 days (24–211 days) in NHospS patients. The main reasons for unplanned readmission were similar for both HospS and NHospS patients, and the three main reasons were infectious diseases (36.2%), cerebrovascular diseases (16.4%)—particularly IS recurrence (10.5%), and cardiovascular diseases (10.5%). The median in-hospital length of stay of readmissions was 9 days (interquartile range: 4–20 days), and 27 (17.6%) patients died during readmission.

**Table 3:** Characteristics and causes of patients' all-cause readmissions within 1 year (*n* = 152).

	**Overall**		**HospS**		**NHospS**		***p*-Value**
	***n***	**%**	***n***	**%**	***n***	**%**	
One-year all-cause readmissions	152	23.7	101	26.0	51	19.9	0.077
0–30 days	46	30.3	32	31.7	14	24.5	
31–180 days	65	42.8	43	42.6	22	43.1	
181–365 days	41	26.9	26	25.7	15	26.4	
Median hospital readmission stays (IQR), days	9	(4–20)	7	(3–17)	9	(5–16)	0.490
Readmission case fatality^*^	27	17.6	19	18.8	8	15.7	0.634
Causes of unplanned readmissions							
Infectious diseases	55	36.2	40	39.6	15	29.4	0.217
Respiratory tract	23	41.8	17	42.5	6	40.0	
Urinary tract	15	44.4	12	50.0	3	20.0	
Sepsis	11	27.3	7	6.9	4	26.7	
Other	6	10.9	4	25.0	2	13.3	
Cerebrovascular disease (IS)	25 (16)	16.4 (10.5)	16 (10)	15.8 (9.9)	9 (6)	17.6 (11.5)	0.777
Cardiovascular disease (MI)	16 (8)	10.5 (5.3)	9 (3)	8.9 (3.0)	7 (5)	13.7 (9.8)	0.361
Neoplasm	9	5.9	8	7.9	1	2.0	0.142
Gastrointestinal diseases	11	7.2	5	4.9	6	11.7	0.126
Chronic respiratory diseases	6	3.9	4	4.0	2	3.9	0.678^a^
Other	30	19.7	19	18.8	11	21.6	0.687
Stroke-related readmission^‡^	82	53.9	54	53.5	28	54.9	0.867
0–30 days	25	30.5	17	31.5	8	28.6	
31–180 days	37	45.1	26	48.1	11	39.3	
181–365 days	20	24.4	11	20.4	9	32.1	

*HospS, hospitalized stroke patients; NhospS, non-hospitalized stroke patients; IS, ischemic stroke; MI, myocardial infarction.*

* *Readmission case.*

† *Includes myocardial infarction, arrhythmia, congestive heart failure, and valvular disease.*

‡ *Includes recurrent vascular events and complications that warranted readmission, including stroke, pneumonia, urinary tract infection, peripheral and coronary artery disease, hip fracture, and pulmonary embolism.*

a * Fisher exact test*.

### Stroke-Related Readmissions

Stroke-related readmissions represented 53.9% of the total, with similar proportions in both HospS and NHospS patients (Table 3).

### Factors Associated With Readmission or Death

Table 4 shows the univariable and multivariable analyses for all-cause readmission or death within 1 year. In the multivariable regression analysis, male sex, age, pre- and post-stroke functional status, and diabetes were independent factors of readmission/death within 1 year. Having had a lacunar stroke (hazard ratio, 0.52; 95% CI, 0.31–0.87) and getting medical attention in <24 h after stroke symptoms (hazard ratio 0.61; 95% CI, 0.42–0.87) improved the odds of not being readmitted or death within 1 year, in this analysis.

**Table 4:** Univariable and multivariable readmission/death analyses.

	**Univariable**		***p*-Value**	**Multivariable**		***p*-Value**
	**HR**	**95%CI**		**HR**	**95%CI**	
Men vs. women	0.86	0.64–1.14	0.287	1.54	1.02–2.32	**0.039**
Age, years	1.05	1.03–1.06	** <0.001**	1.04	1.02–1.05	** <0.001**
Hospitalized (Yes vs. No)	1.41	1.04–1,90	**0.027**	1.39	0.98–1.96	0.062
Lacunar stroke vs. others	0.35	0.21–0.57	** <0.001**	0.52	0.31–0.87	**0.012**
Pre-stroke mRS ≥ 2 vs. mRS <2	2.73	2.05–3.63	** <0.001**	1.61	1.17–2.21	**0.003**
Pre-existing comorbidities (Yes vs. No)						
Hypertension	1.68	1.14–2.47	**0.008**	1.17	0.78–1.76	0.450
Diabetes mellitus	1.61	1.19–2.17	**0.002**	1.52	1.11–2.09	**0.010**
Atrial fibrillation	2.09	1.53–2.85	** <0.001**	1.16	0.81–1.65	0.418
Congestive heart failure	1.87	1.36–2.56	** <0.001**	1.20	0.85–1.68	0.302
Myocardial infarction	0.98	0.57–1.61	0.937	0.68	0.40–1.14	0.141
Hypercholesterolemia	0.93	0.68–1.20	0.484	0.98	0.72–1.33	0.890
Smoking	0.61	0.44–0.84	**0.002**	0.68	0.44–1.06	0.085
Dementia	1.90	1.32–2.74	**0.001**	1.02	0.68–1.52	0.931
Neoplasm	1.91	1.35–2.07	** <0.001**	1.36	0.95–1.95	0.094
Post-stroke mRS ≥ 2 vs. mRS <2	4.91	2.67–9.03	** <0.001**	2.72	1.44–5.13	**0.002**
Previous admissions (Yes vs. No)	2.02	1.35–3.02	**0.001**	1.35	0.88–2.08	0.163
Medical attention <24 h vs. >24 h	0.77	0.56–1.11	0.174	0.61	0.42–0.87	**0.007**

*mRS, modified Rankin Scale.*

*Bold entries indicate statistical significance (p < 0.05)*.

## Discussion

This research provides novel information about hospitalization patterns and readmissions/death in HospS and NHospS patients in Portugal, reinforcing the current knowledge on this issue. In our population-based study, we found that 35.7% of stroke patients were not hospitalized at stroke presentation and that 33.0% of HospS patients and 24.9% of NHospS patients were readmitted/deceased within 1 year. The leading causes of readmission in both cohorts were infections, recurrent stroke, and cardiovascular diseases. Stroke-related readmissions were observed in more than half of the patients in both groups. Male sex, age, pre- and post-stroke mRS ≥2, and diabetes were independent factors of readmission/death within 1 year. In contrast, having a lacunar stroke and getting medical assistance in <24 h improved the chances of not being readmitted/deceased within 1 year.

### Hospitalization Rate

Our estimates of non-hospitalizations (35.7%) at stroke presentation are in the upper range to what was previously reported in similar studies (4, 21); this may reflect different study methodologies but also differences between regions and countries regarding stroke referral patterns and care pathways (21). Moreover, patients' characteristics could explain the differences since, for instance, the NHospS patients were older, had fewer cases of hemorrhagic stroke and more of lacunar stroke, had a lower post-stroke disability, had a lower median NIHSS, and were more prone to seeking medical attention later than 24 h than the HospS cohort. As we have stated elsewhere (22), these patient characteristics might have led to a vascular study in the ED and a decision for additional and prompt outpatient management. Also, a few other patients may have been transferred to other hospitals and, therefore, not counted as admissions.

We understand that other factors may have affected the hospitalization decision, including the patient's or their caregiver's choice, policies of local stroke services, and resource availability (4). Also, hospitalization of all stroke patients is not practical or feasible. Since stroke is a rather heterogeneous disease that includes distinct types, having different prevalence and incidence rates, risk factors, and management guidelines that may lead to different outcomes and disease burdens, it is important to select the most appropriate stroke care setting in order to improve prognosis and limit costs (8, 23).

### Readmission/Death Rates

We found that 33.0% of HospS and 24.9% of NHospS patients were readmitted/deceased within 1 year. Comparable studies showed similar results in hospitalized patients, reporting readmission/death rates between 13 and 62% (24–27), but few address this outcome in both ischemic and hemorrhagic strokes (25–27). To our knowledge, no studies in the literature have addressed the 1-year readmission/death rates in NHospS patients with all stroke types. Still, one study that only includes minor IS reported a readmission/death rate at 30 days in NHospS (8) similar to ours.

Our data showed that HospS patients were more likely to be readmitted or deceased within 1 year than NHospS patients, even though HospS patients could have had early access to secondary prevention measures and rehabilitation. The poorer outcome in the HospS group might be explained by important differences in clinical characteristics, namely, HospS patients had more cardiac comorbidities (namely, atrial fibrillation), worse post-stroke functional status, and different stroke subtype/etiology (less lacunar/small artery occlusion strokes) than NHospS patients, and previously published studies have associated those factors with in-hospital worse stroke outcome/morbidity and readmission risk factors (7, 28–30). Furthermore, hospitalization has associated inherent hazards and heightened risks (4) that may have contributed to a worse functional status at discharge in HospS patients, a net effect of pre-existing comorbidities, stroke severity, and in-hospital complications, known to be linked to a higher risk of readmission (28).

On the other hand, NHospS patients may have benefited from a prompt follow-up since they were reassessed within 1 month in the outpatient stroke clinic, thus mitigating the lack of a timely intervention due to possible delays in appointment times (10, 31). However, contrary to other studies (4, 32), being hospitalized, *per se*, was not found to be a detrimental factor for readmission/death after adjusting for many of the case-mix variables, such as age, sex, pre-stroke disability, comorbidities, stroke subtype (dichotomized in lacunar vs. non-lacunar), and post-stroke disability (post-stroke mRS at hospital admission), as suggested by Davenport et al. (33).

Furthermore, these results may be attributable to patient selection and referral patterns and do not support stroke hospitalization eviction (4, 34). We agree, according to the best evidence (35), that as many acute stroke patients as possible should be hospitalized and treated within a stroke unit; nevertheless, our study questions the feasibility of a prompt stroke outpatient management for certain types of stroke, as it is elsewhere considered (4, 8, 9). In these cases, expedited access to a stroke specialized outpatient clinic should be warranted (4, 31).

### Readmission Causes and Stroke-Related Readmissions

During the first year after stroke, infections, recurrent stroke, and cardiovascular events were the leading causes of readmission in both HospS and NHospS cohorts. Disability, age, sex, and vascular risk factors are recognized predictors of post-stroke infections (7, 36), yet, we did not find differences in the incidence of infections in both groups. Our proportion of readmissions due to cerebro-cardiovascular causes confirms that the vascular risk remains elevated in the long-term and that stroke may be a sentinel event that confers the longstanding risk of adverse outcomes, stressing the need for long-term risk reduction management strategies in both cohorts (37). Apart from the aforementioned readmission causes, our study found that other non-vascular diseases are also prevalent causes of stroke readmission. This information may help healthcare providers determine the risk of new diseases after a stroke that may lead to re-hospitalization (7).

Stroke-related readmission incidence, temporal pattern, and causes were similar in both cohorts, emphasizing the importance of stroke secondary prevention measures and the need for a better understanding of the causes and reasons that mediate this type of readmissions (38, 39).

### Factors Associated With Readmission or Death

Several well-recognized and potentially preventable stroke risk factors, such as hypertension, diabetes, atrial fibrillation, and congestive heart failure, are associated with readmission/death and considered risk factors (25–27). This finding stresses the need for a careful follow-up and coordinated, well-structured multifaced interventions, such as those depicted in the STROKE-CARD care and INSPiRE-TMS studies, increasing the stroke patients' adherence to the recommended secondary stroke preventive measures (40, 41).

Our study's multivariable analyses consubstantiate that age, male sex, comorbidities (such as diabetes), and pre- and post-stroke worse functional status are risk factors for readmission (25–27). Identifying these explicit risk factors, which can also contribute to a patient's frailty and consequently increase the risk of death/institutionalization (42), may help implement targeted interventions to reduce readmissions/death at a population level.

In our study, lacunar strokes had a decreased risk of readmission/death, as found by others (7, 28, 43). Common risk factors between post-stroke readmission and death could explain these results (44). Furthermore, lacunar stroke may be related to fewer post-stroke complications, such as dysphagia or sphincter dysfunction, which are well-known risk factors for infections (28)—the more prevalent cause of readmission found.

Seeking medical attention within 24 h after a stroke improved the odds of not being readmitted/deceased within 1 year. Indeed, the most beneficial effect on a stroke patient's disability comes from early hospital arrival, highlighting the impact of stroke management during the acute phase, including the implementation of Stroke Code pathways and stroke units, where IS and ICH may equally benefit from early intervention by trained staff (23). This finding reinforces the importance of stroke awareness campaigns to educate the population on the recognition and surveillance of stroke warning signs and the importance of an early arrival to the hospital after a stroke (8, 45).

## Limitations

This study has some limitations. Firstly, the data between 3 months and 1 year after stroke were collected retrospectively; however, we tried to temper this issue by acquiring information from medical records instead of only from administrative data. Secondly, the accuracy of our results may have been impaired by our definition of vascular risk factors; for instance, patients were considered non-smokers if no information about their smoking habits was known.

In a small subset of patients of the study at the Centro Hospitalar Universitário do Porto, the hospitalization decision may have been influenced by clinical staff knowing that including patients in the stroke incidence study would lead to fast assessment in the project specialized outpatient clinic. After the years 2009–2012, when our data were collected, significant changes were observed in clinical stroke guidelines, particularly endovascular stroke treatment, and our results do not account for that. Nonetheless, few reports address readmissions of all stroke subtypes in non-hospitalized patients, and to our knowledge, this is the first time that these data are described in Portugal.

We did not include data about hospitalization complications or, in both cohorts, information about stroke secondary prevention patterns of care (i.e., anticoagulation, antihypertensive, or lipid-lowering therapy), known factors of stroke quality of care (34) that may have contributed to some of the observed differences in both groups. Lastly, our proportion of readmission may have been underestimated by not collecting data directly from private hospitals, although this information was mostly posteriorly registered in the patient's NHS medical records to which we have had access.

## Conclusion

We have found that one-third of stroke patients were not hospitalized, and the readmission/death rate was higher in the HospS patients. Still, the readmission/death incidence difference was likely due to other factors than hospitalization itself. Our stroke patients had had different clinical processes of care, but we have not encountered a different pattern of readmission causes in both cohorts, emphasizing the need for tailored individual interventions in stroke patients to prevent readmissions. Our results provide important information about patient-level characteristics and readmissions in HospS and NHospS patients, which may help implement targeted health-related policies to reduce the burden of stroke and its complications.

## Data Availability Statement

The data analyzed in this study was obtained from the second population-based register undertaken in Northern Portugal (ACIN2), the following licenses/restrictions apply: permission to access the data must be granted by the main ACIN2 investigators. Requests to access these datasets should be directed to Pedro Abreu, pmabreu@netcabo.pt.

## Ethics Statement

The studies involving human participants were reviewed and approved by Ethics Committee of the Centro Hospitalar Universitário de São João and the Centro Hospitalar Universitário do Porto. The patients/participants provided their written informed consent to participate in this study. This study was conducted in accordance with the World Medical Association Declaration of Helsinki.

## Author Contributions

PA was responsible for the study conceptualization, data acquisition, part of the statistical analysis and table elaboration, and drafting of the main manuscript. RM was the main responsible for the statistical analyses and table and figures elaboration, and contributed to the study conceptualization/methodology, data acquisition, and article draft/review. DB contributed to the data acquisition and study conceptualization. EA and MC critically reviewed this article and contributed to the study conceptualization/methodology and article draft. All authors contributed to the article and approved the submitted version.

## Conflict of Interest

The authors declare that the research was conducted in the absence of any commercial or financial relationships that could be construed as a potential conflict of interest.

## Publisher's Note

All claims expressed in this article are solely those of the authors and do not necessarily represent those of their affiliated organizations, or those of the publisher, the editors and the reviewers. Any product that may be evaluated in this article, or claim that may be made by its manufacturer, is not guaranteed or endorsed by the publisher.

## Abbreviations

FELS: first-ever-in-a-lifetime stroke
NHospS: non-hospitalized stroke
HospS: hospitalized stroke
ED: emergency department
NHS: National Health Service
IS: ischemic stroke
ICH: intracerebral hemorrhage
SAH: subarachnoid hemorrhage
NIHSS: National Institutes of Health Stroke Scale
mRS: modified Rankin Scale
SD: standard deviation
IQR: interquartile range
MI: myocardial infarction.
